# Identification of a Protein Network Driving Neuritogenesis of MGE-Derived GABAergic Interneurons

**DOI:** 10.3389/fncel.2016.00289

**Published:** 2016-12-21

**Authors:** Sira A. Franchi, Veronica Astro, Romina Macco, Diletta Tonoli, Jean-Vianney Barnier, Martina Botta, Ivan de Curtis

**Affiliations:** ^1^Cell Adhesion Unit, Division of Neuroscience, San Raffaele Scientific Institute and San Raffaele UniversityMilano, Italy; ^2^Neuroscience Paris-Saclay Institute, UMR 9197, Centre National de la Recherche Scientifique-Université Paris-SudOrsay, France

**Keywords:** GABAergic interneurons, growth cones, neurites, Rac GTPases, scaffold proteins

## Abstract

Interneurons are essential modulators of brain activity and their abnormal maturation may lead to neural and intellectual disabilities. Here we show that cultures derived from murine medial ganglionic eminences (MGEs) produce virtually pure, polarized γ-aminobutyric acid (GABA)-ergic interneurons that can form morphologically identifiable inhibitory synapses. We show that Rac GTPases and a protein complex including the GIT family scaffold proteins are expressed during maturation *in vitro*, and are required for the normal development of neurites. GIT1 promotes neurite extension in a conformation-dependent manner, while affecting its interaction with specific partners reduces neurite branching. Proteins of the GIT network are concentrated at growth cones, and interaction mutants may affect growth cone behavior. Our findings identify the PIX/GIT1/liprin-α1/ERC1 network as critical for the regulation of interneuron neurite differentiation *in vitro*, and show that these cultures represent a valuable system to identify the molecular mechanisms driving the maturation of cortical/hippocampal interneurons.

## Introduction

The γ-aminobutyric acid (GABA)-ergic interneurons are essential modulators of brain activity (Gelman and Marín, 2010), and their abnormal maturation may alter the balance between excitatory and inhibitory brain activity leading to neural and intellectual disabilities (Brooks-Kayal, 2011). In this direction, different studies on the mechanisms underlying different mental disorders including intellectual disability, schizophrenia, autism-related disorders, and attention deficit hyperactivity disorder (ADHD) have identified defects in the cortical GABAergic interneurons that may importantly contribute to the pathogenesis of these diseases (Kitamura et al., 2002; Lewis et al., 2005; Orekhova et al., 2007; Lawrence et al., 2010; Sebe and Baraban, 2011; Won et al., 2011).

Most cortical and hippocampal GABAergic interneurons are born in the caudal and medial ganglionic eminences (CGE and MGE, respectively), which are transitory embryonic structures in the ventral telencephalon (Wonders and Anderson, 2006). Interneurons migrate to populate cortex and hippocampus (Guo and Anton, 2014). After reaching their destination, GABAergic interneurons make synaptic connections with excitatory neurons or other interneurons. Several extracellular cues and transcription factors have been identified as essential players required for the differentiation of cortical and hippocampal GABAergic interneurons, while much less is known about the intracellular mechanisms driving the post-migratory development of these cells (Hernández-Miranda et al., 2010). In this respect, recent results have shown that the two Rac GTPases expressed during neuronal development, Rac1 and Rac3, are important for the development of MGE-derived cortical and hippocampal GABAergic interneurons (de Curtis, 2014). Rac1 and Rac3 play a pivotal role in the maturation of MGE-derived cortical and hippocampal interneurons, since the deletion of either Rac1 or Rac3 has negative effects on the maturation and formation of inhibitory synapses by GABAergic cells, with behavioral consequences that include generalized hyperactivity, and cognitive as well as functional impairment of the inhibitory circuits (Pennucci et al., 2016).

Interestingly, a number of genes that are functionally linked to Rac GTPases are mutated in human patients with intellectual disabilities, supporting the idea that pathways linked to Rac GTPases are important for normal brain development and cognitive functions. Among the genes affected in human patients are components of a protein network including GIT scaffold proteins with ARF-GAP activity (Hoefen and Berk, 2006), PIX guanine nucleotide exchange factors (GEFs) for Rac/Cdc42, and PAK kinases. Members of these families are implicated in cognitive defects in humans, since the genes for the GEF αPIX (*ARHGEF6*) and PAK3 (*PAK3*) are mutated in patients with X-linked intellectual disability (Allen et al., 1998; Kutsche et al., 2000), while the gene for GIT1 (*GIT1*) is altered in patients with attention deficit hyperactivity disorder (ADHD) (Won et al., 2011). Published data support a role of PAK3, αPIX, and GIT1 in the formation and plasticity of excitatory synapses (Boda et al., 2004; Meng et al., 2005; Zhang et al., 2005; Nodé-Langlois et al., 2006; Kreis et al., 2007; Thévenot et al., 2011; Ramakers et al., 2012), while the implication of this protein network in the development of GABAergic interneurons remains to be established. In this direction, PAK3 has been shown to be upregulated during the postmigratory development of GABAergic interneurons (Cobos et al., 2007), while the knockout of GIT1 induces a specific loss of GABAergic synaptic terminals in the hippocampus, suggesting a role for these proteins in the development of interneurons (Won et al., 2011).

The possibility to establish cultures of pure interneurons would allow the analysis of the implication of specific molecules in the maturation of GABAergic cells *in vitro*, with the aim of identifying signaling pathways required for the formation of functional inhibitory circuits. Previous studies have used explants from MGE or short-term cultures of MGE-derived cells to address the role of different cues and their receptors in the migration and development of interneurons (Pozas and Ibáñez, 2005; Zimmer et al., 2008; Li et al., 2012; Villar-Cerviño et al., 2015). Still, a detailed analysis of the development of these cells *in vitro* is lacking. In this study, we describe the set up and characterization of a culture system of MGE-derived interneurons that recapitulates the maturation of interneurons from the extension of neurites and the formation of distinguishable axon and dendrites, to the formation of morphologically identifiable GABAergic synapses. We have used these cultures to show the requirement of Rac GTPases for the maturation of neurites by MGE-derived interneurons. We have then extended the molecular analysis to define the role of the PIX/GIT protein network linked to Rac GTPases in the development of these cells. The analysis performed either by silencing the endogenous proteins, or by expressing GIT1 mutants affecting the activation or the interaction with distinct partners has highlighted the importance of the PIX/GIT network in the maturation of MGE-derived interneurons.

## Results

### MGE-derived cells mature into GABAergic interneurons *in vitro*

**Figure 1:**
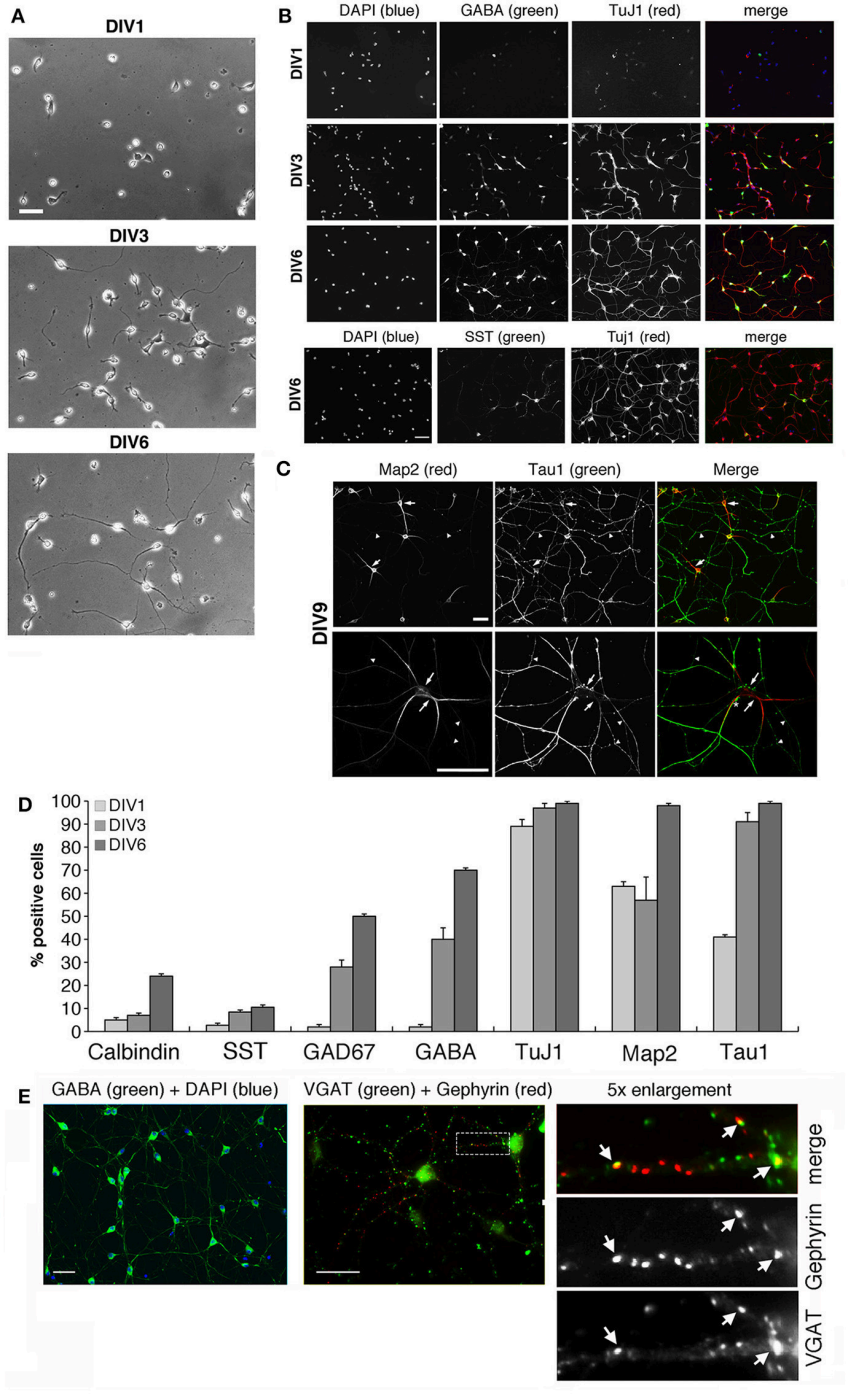
**Morphological maturation of MGE-derived interneurons**. See also Figure S1. **(A)** Differentiation of MGE-dissociated cells from E14.5 mouse embryos plated on coverslips with PLL and laminin-1, and fixed at (DIV1), DIV3, or DIV6. Scale bar, 40 μm. **(B–D)** Maturation of MGE-derived GABAergic interneurons *in vitro*. **(B)** Increasing expression of neuronal and GABAergic markers detected by immunofluorescence. Scale bar, 40 μm. **(C)** Localization of tau-positive axons (arrowheads) and MAP2-positive dendrites (arrows) in DIV9 interneurons. The asterisk in the merge of the lower panel shows an example of a tau-positive axon running parallel and very close to a MAP2-positive dendrite. Scale bar, 40 μm. **(D)** Quantification of the percentage of cells positive for the indicated neuronal markers at DIV1–DIV6 (*n* = 368 for calbindin and GAD67; *n* = 186 for GABA; *n* = 193 for TuJ1; *n* = 74 for MAP2 and tau; *n* = 71 for somatostatin, SST). **(E)** Left: by DIV15 most cells are positive for GABA. Center: immunostaining of DIV20 interneurons for VGAT and gephyrin. Right: 5-fold enlargement of the boxed region to show morphologically defined inhibitory synapses identified by the juxtaposition of VGAT-positive presynaptic terminals with gephyrin-positive postsynaptic terminals (arrows). Scale bars, 40 μm.

We have optimized the protocol for primary cultures of murine GABAergic interneurons obtained from E14.5 MGEs, a useful and simplified system to investigate the machinery required for different steps of interneuron development *in vitro*. Several extracellular matrix proteins were tested as substrates to optimize the maintenance and differentiation of interneurons *in vitro*. Type I and IV collagens were poor substrates for these cells (not shown), while fibronectin and laminin-1 facilitated adhesion of MGE-derived cells at day 1 *in vitro* (DIV1), but at DIV2 the cells already started to detach from substrates coated with either glycoprotein (not shown). MGE-derived cells clustered on Matrigel-coated substrates, while on 200 μg/ml poly-L-lysine (PLL) most MGE-derived cells detached by DIV2-DIV3. Best substrates were obtained by coating plastic or glass with 200 μg/ml PLL followed by 20 μg/ml laminin-1. This coating allowed MGE-derived precursors to attach and develop up to DIV6 (Figure 1A) and later. The differentiation of MGE-derived cells into GABAergic interneurons *in vitro* was evaluated by immunofluorescence on cells at different DIVs analyzed for the expression of the neuronal markers Tuj1, MAP2 and tau, and of the specific GABAergic markers calbindin, somatostatin, GAD67 and GABA (Figures 1B,C). Under these conditions neurons extend an elaborated network of neurites, with clear distinction between MAP2-positive dendrites and tau1-positive axons from DIV9 on (Figure 1C, Figure S1A), indicating that the MGE-derived cells could develop neuronal polarity under these conditions. An increase in signal intensity and in the proportion of cells expressing these markers was observed between DIV1 and DIV6 (Figure 1D). At DIV6 virtually all cells were positive for TuJ1, MAP2 and tau. Moreover 71% of the neurons became positive for GABA, 50% for GAD67, 22% for calbindin, and 10% for somatostatin, indicating that most MGE-derived cells showed a GABAergic phenotype *in vitro*.

Interestingly, long-term cultures were achieved by seeding the MGE-derived cells on Matrigel (2.5 mg/ml). By DIV15 virtually all cells were positive for GABA, and between DIV9 and DIV20 inhibitory synapses were identified morphologically by the apposition of the presynaptic marker VGAT with postsynaptic gephyrin (Figure 1E), a protein required for clustering postsynaptic GABA receptors (Sassoè-Pognetto et al., 2000). Altogether our data indicated that MGE-derived GABAergic interneurons may represent an optimal system to address the molecular mechanisms underlying different phases of GABAergic differentiation *in vitro*.

A critical aspect in our cultures was the variability in cell survival among different preparations. The brain derived neurotrophic factor (BDNF) promotes the development of the GABAergic phenotype, neuritic branching, and cell viability in MGE-derived cultures (Pozas and Ibáñez, 2005). Addition of 50 ng/ml BDNF to DIV1 cultures improved reproducibility by decreasing the variability among preparations, and enhanced the development of neurites (Figure S1B). BDNF-treated interneurons showed a 20% increase of primary dendrites compared to untreated cells (Figure S1C), and an enhanced axonal network (Figure S1D). Moreover, addition of BDNF to primary cultures increased cell survival, especially after transfection (next sections).

### Rac GTPases are required for the development of gabaergic interneurons

**Figure 2:**
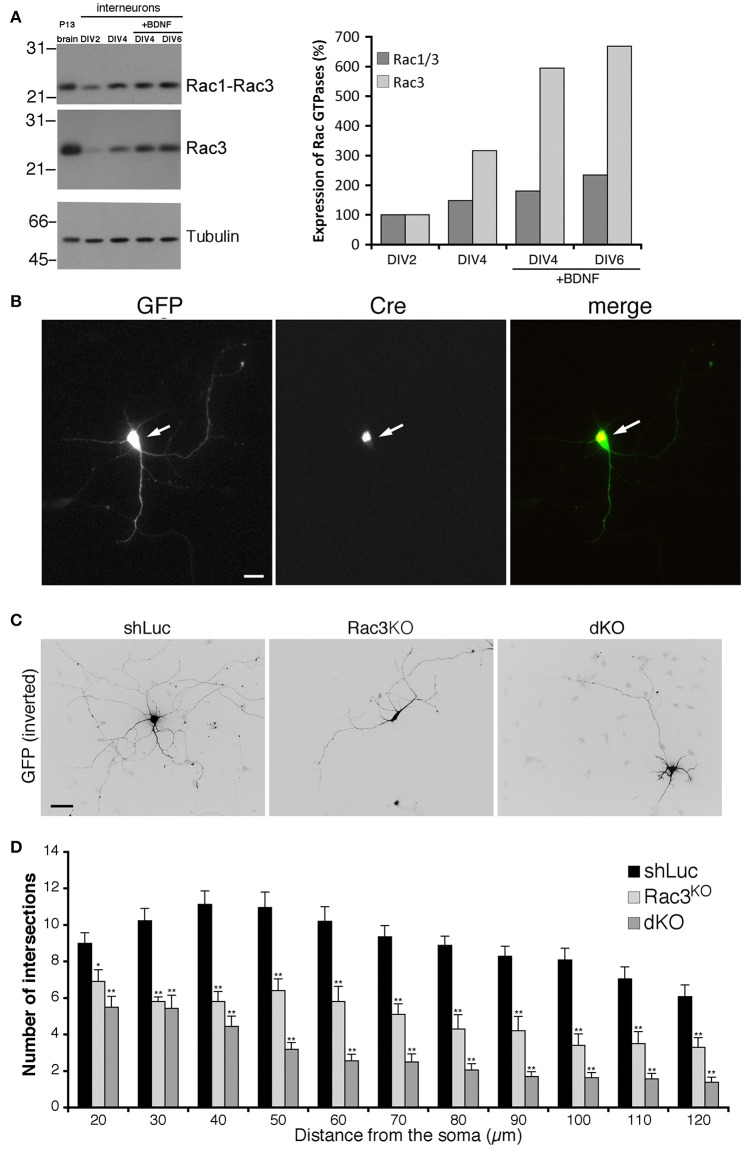
**Rac depletion inhibits neurites development in MGE-derived interneurons. (A)** Immunoblot for Rac, Rac3, and tubulin on lysates from P13 brain or MGE-derived cultures, with or without BDNF. Filters were incubated for Rac3, stripped, and reprobed for Rac (both Rac1 and Rac3). Graph on the right: quantification from the blots. Values normalized to tubulin were compared to the expression at DIV2 taken as 100%. **(B)** MGE-derived Rac1^F/F^//Rac3^KO^ cells cotransfected at DIV1 with GFP and GFP-Cre. The arrows show a cell expressing the Cre protein. Scale bar, 20 μm. **(C)** MGE-derived interneurons transfected at DIV1 and cultured with BDNF until DIV6; immunostaining for GFP. Bar, 40 μm. **(D)** Sholl analysis at DIV6 on control MGE-derived cells (shLuc), and on Rac1^F/F^//Rac3^KO^ MGE-derived cells transfected with either GFP (Rac3^KO^) or GFP-Cre (dKO) (*n* = 25 neurons for shLuc, 16 dKO neurons, 10 Rac3KO neurons). ^*^p < 0.05, ^**^p < 0.005; Student's *t*-test.

Rac1 and Rac3 GTPases are co-expressed in mammalian central nervous system. Our previous works has shown that Rac1 represented approximately 92% and Rac3 8% of total Rac proteins in P7 mouse brain (Corbetta et al., 2009). Analysis *in vivo* has shown that single and double Rac knockout affects the development of cortical and hippocampal interneurons (Vaghi et al., 2014; Tivodar et al., 2015; Pennucci et al., 2016). Here we have tested the effects of Rac deletion on the maturation of MGE-derived interneurons *in vitro*. Immunoblotting with an anti-Rac antibody recognizing both proteins, or with an antibody specific for Rac3 showed that the expression of both proteins increased with time in MGE-derived cultures (Figure 2A). Rac3 levels in presence of BDNF increased 6-folds between DIV2 and DIV4. We then evaluated the effects of Rac depletion on interneuron maturation. For this, MGE-derived cells with double or single Rac3 knockout were compared with control cells for neuritogenesis. Double knockout interneurons were obtained by transfecting DIV1 MGE-derived cells from Rac1^F/F^//Rac3^KO^ mice with pEGFP-Cre to inactivate the *Rac1* gene (Corbetta et al., 2009). The morphology of these cells was compared to cells from Rac1^F/F^//Rac3^KO^ mice transfected with GFP alone (Rac3^KO^ cells), or to wildtype neurons transfected with a control plasmid (Figure 2B). Sholl analysis revealed a reduction of neuritic branching in Rac3^KO^ cells, and an even stronger reduction in double knockout cells (Figures 2C,D). Therefore, both endogenous Rac proteins are involved in the development of the neuritic tree of GABAergic cells.

### A protein network functionally linked to Rac GTPases drives dendritic maturation in gabaergic interneurons

**Figure 3:**
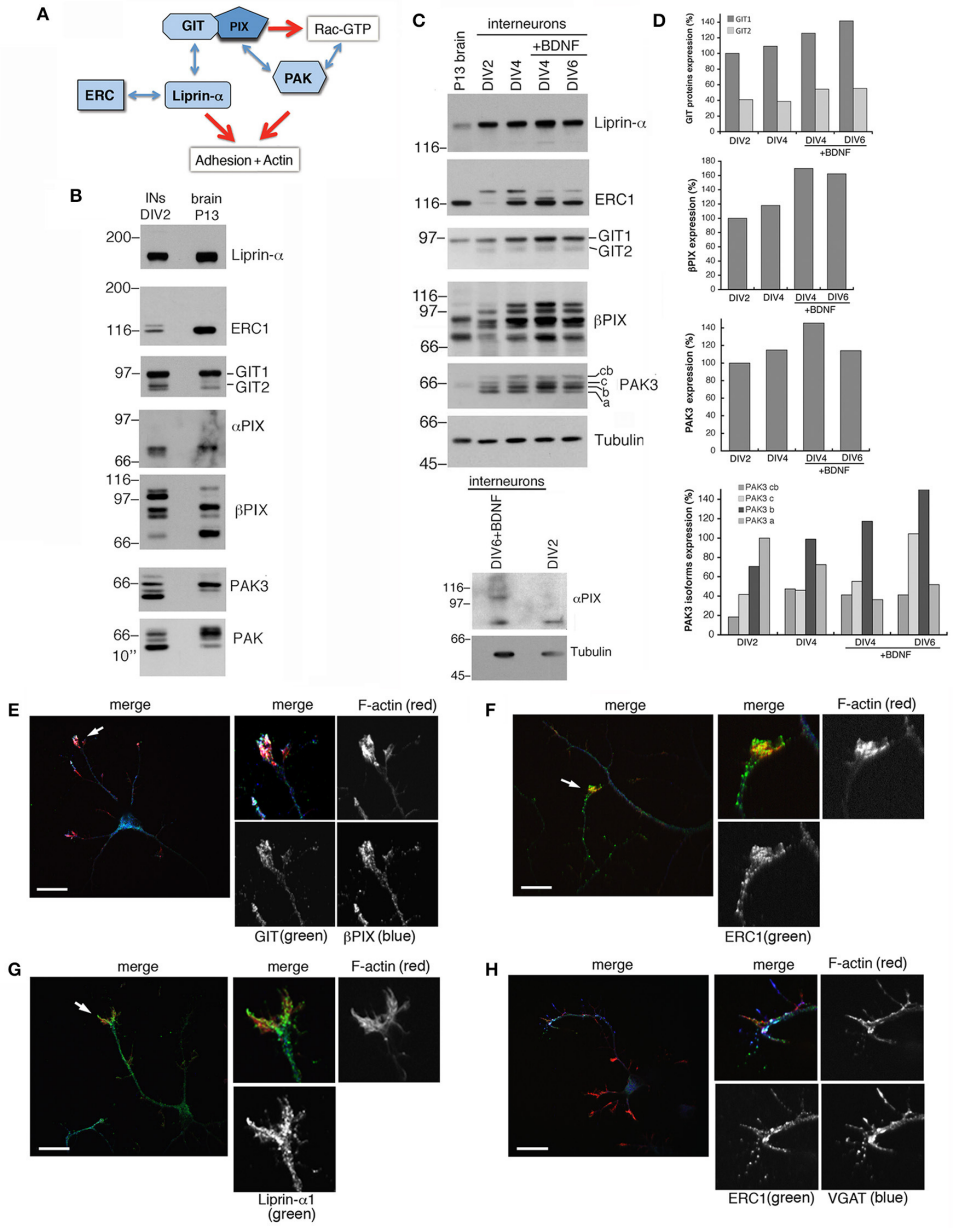
**Several components of the PIX/GIT protein network are increasingly expressed during interneuron development *in vitro***. **(A)** Protein network analyzed in this study. GIT/PIX complexes may activate Rac's via the GEF activity of PIX. Rac-GTP may then interact with effector kinases of the PAK family. On the other side GIT proteins may interact with the liprin-α1/ERC1 complex regulating cell adhesion and motility. **(B)** Immunoblotting for the indicated proteins in DIV2 interneurons and P13 brain lysate (80 μg of protein per lane). **(C)** Immunoblots for liprin-α1, ERC1, GIT, PIX, PAK3, and Rac proteins in MGE-derived interneurons maturing *in vitro*. On the left lane a lysate from P13 brain is shown for comparison (50 μg of protein per lane). MGE-derived cells were cultured up to DIV2–DIV6 with or without BDNF (added at DIV1). Expression of αPIX is very low. **(D)** Quantification of the relative levels of the proteins in lysates from cultures at the indicated stage, ±BDNF. Values were normalized to the respective tubulin. **(E–H)** Immunostaining on DIV5/DIV6 MGE-derived interneurons to detect the localization of endogenous GIT and βPIX **(E)**, ERC1 **(F,H)**, and liprin-α1 **(G)**. Evident colocalization of GIT with βPIX **(E)**, and of ERC1 with VGAT **(H)**. On the right of each panel: 2.5-fold enlargements of regions indicated by arrows. Scale bars, 20 μm.

Protein complexes including Rac activators of the PIX family and their binding partners of the GIT and PAK families (Figure 3A) form stable complexes that can be isolated from brain and cell lysates (Totaro et al., 2007, 2012). These complexes interact specifically with active GTP-Rac's (Di Cesare et al., 2000). The scaffold proteins of the GIT family and the Rac GEFs of the PIX family are involved in neuronal development (Albertinazzi et al., 2003; Zhang et al., 2005; Totaro et al., 2012). Both GIT family proteins (GIT1 and GIT2), and PIX family proteins (αPIX and βPIX), as well as different PAK proteins including PAK3 were expressed in DIV2 MGE-derived cells (Figure 3B). Previous work has indicated that the increased expression of the PAK3 kinase correlates with the maturation of GABAergic interneurons (Cobos et al., 2007). Using PAK3 antibody on lysates from the MGE-derived cultures all four protein isoforms were detected: *cb* (72 KDa), *c* (69 KDa), *b* (68 KDa) and *a* isoform (65 KDa) (Kreis et al., 2008). As described (Cobos et al., 2007), the expression of PAK3 in MGE-derived cells increased with time in culture (Figures 3C,D). The PAK3*b* isoform was the most expressed in MGE-derived neurons, and with the exception of PAK3*a*, the expression of the other isoforms increased during development. The elevated expression of GIT1, βPIX, and PAK3 molecules in GABAergic interneurons and their increase during development *in vitro* supported their possible implication in the differentiation of MGE-derived interneurons *in vitro*. In addition, comparison of MGE-derived DIV2 lysates with P13 brain lysates showed distinct patterns of expression for PAK, PAK3, and βPIX. Two other components of this protein network (Figure 3A), the GIT-interacting protein liprin-α1 and its binding partner ERC1/ELKS, which regulate efficient synaptic transmission (Südhof, 2012; Kittelmann et al., 2013) and non-neuronal cell motility (Astro and de Curtis, 2015), were also detected in MGE-derived cells (Figures 3B,C). Immunofluorescence on DIV5/DIV6 interneurons showed that GIT, PIX, liprin-α1 and ERC1 proteins were expressed in cultured interneurons and accumulated in growth cones (Figures 3E–H). During neuritogenesis, between DIV1 and DIV6, the levels of some of the proteins of the GIT/PIX network increased considerably (Figures 3C,D). Next, we investigated the role of this network in neuritic development.

**Figure 4:**
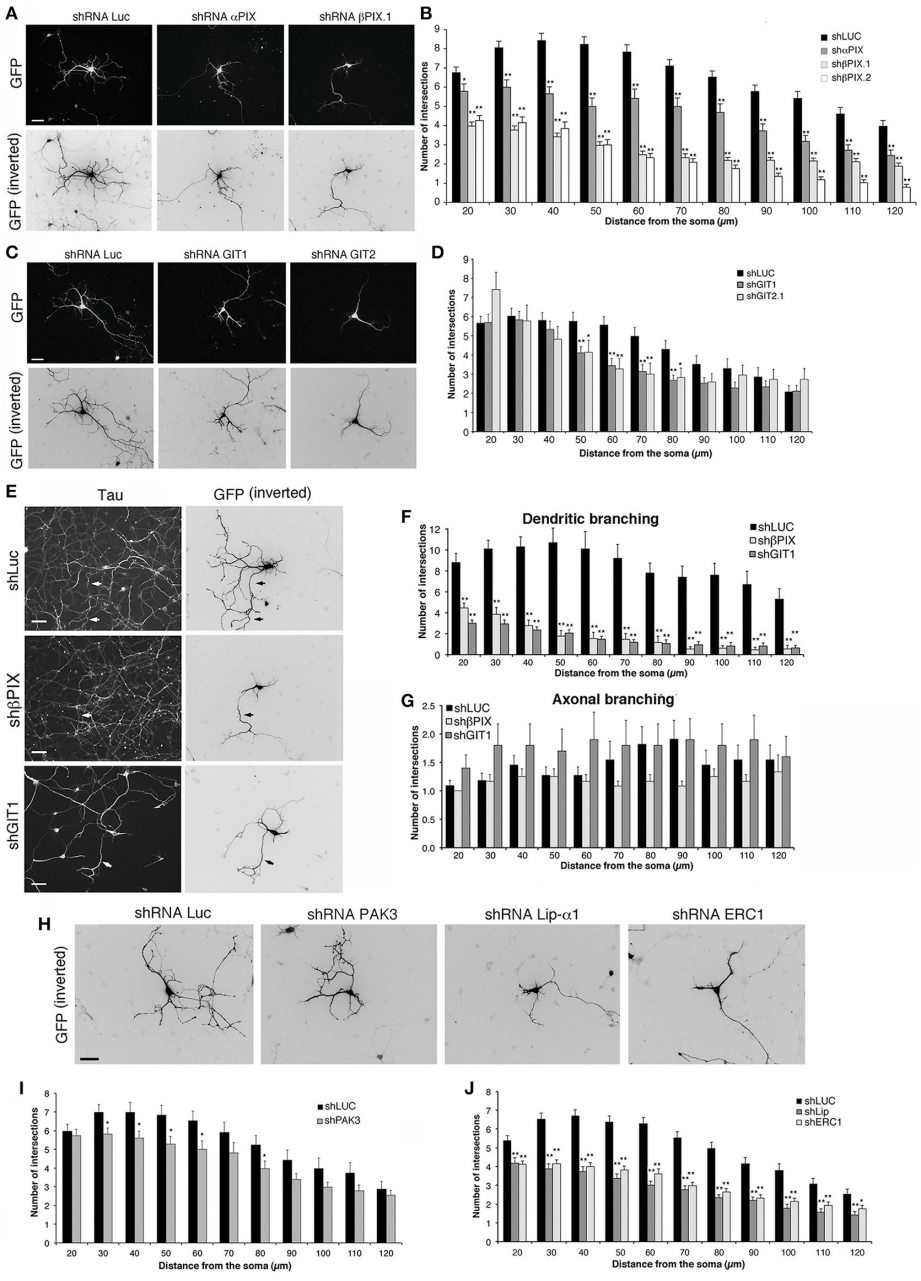
**Down-regulation of proteins of the PIX/GIT complex impairs dendritic development**. See also Figures S2, S3. **(A,C,E,H)** MGE-derived interneurons transfected at DIV1 and cultured with BDNF until DIV6; immunostaining for GFP. **(E)** Neurons immunostained for the axonal marker tau (left) and GFP (right). Arrows show examples of axons costained for tau and GFP. Scale bars, 40 μm. **(B,D,I,J)** Effects of the expression of shRNAs on neurites (Sholl analysis). Bars, means ± SEM. In **(B)**, *n* = 86 neurons for shLuc, 29 neurons for shαPIX; 70 neurons for shβPIX.1, 33 neurons for shβPIX.2; in **(D)** *n* = 41 neurons for shLuc, 36 neurons for shGIT1, 22 neurons for shGIT2; in **(I)** *n* = 34 neurons for shLuc, 44 neurons for shPAK3; in **(J)** *n* = 68 neurons for shLuc, 41 neurons for shLip-α1, 43 neurons for shERC1. ^*^p < 0.05; ^**^p < 0.01. **(F–G)** Effects of the indicated shRNAs on MAP2-positive dendrites **(F)** and tau-positive axons **(G)**. Means ± SEM; *n* = 10–11 neurons for shLuc, 12–13 neurons for shβPIX.1, 10–17 neurons for shGIT1; ^**^p < 0.01.

We used previously characterized targeting sequences and vectors to silence components of the protein network. The efficiency of the shRNAs (short hairpin RNAs) was verified either by silencing the endogenous proteins in transfected mouse NIH-3T3 cells, or by cotransfection of the mouse proteins with the respective silencing vectors into COS7 cells. Silencing was variable for different proteins, ranging from 55 to 70% for overexpressed HA-αPIX and GFP-βPIX, to more than 80–95% for endogenous GIT1, GIT2, liprin-α1, ERC1, and for GFP-PAK3 (Figure S2). αPIX and βPIX are differentially expressed in the brain, where they have been implicated in the maturation of hippocampal neurons (Totaro et al., 2012). Silencing of either αPIX or βPIX by a bicistronic pSUPER-GFP plasmid was used to co-express GFP with specific shRNAs (Figure 4). MGE–derived interneurons were transfected at DIV1 and fixed at DIV6 to identify the transfected GFP-positive neurons for morphological analysis. The down-regulation of βPIX with either one of two different shRNAs caused a strong defect in neurite branching, while silencing of αPIX induced weaker although significant branching defects (Figures 4A,B).

GIT1 and GIT2 are scaffold proteins stably associated with PIX proteins in cells (Totaro et al., 2007) and neural tissues (Totaro et al., 2012). Silencing of GIT proteins verified by transfection into NIH-3T3 of previously characterized shRNAs (Totaro et al., 2012) was very efficient: 84% decrease for GIT1, and 100% decrease for GIT2 (Figure S2). Neurite branching was inhibited by either GIT1 or GIT2 silencing, with stronger inhibition observed after GIT1 silencing (Figures 4C,D). Morphological analysis on neurons immunostained for dendritic and axonal markers showed that silencing of GIT1 and βPIX strongly affected MAP2-positive dendrites (Figure 4F and Figure S3), with no evident effects on axonal branching (Figures 4E,G), indicating that the effects observed on neurite branching were mostly due to defects in dendritic development. We can therefore conclude that the defects observed by Sholl analysis on the neuritic development of interneurons depleted of either βPIX or GIT1 over a range of 120 μm from the cell body largely represent a reduction of dendritic development.

PAK kinases regulate different aspects of neuronal development, including the formation of stable functional excitatory synapses (Boda et al., 2008; Kreis and Barnier, 2009; Dubos et al., 2012). PAK3 is expressed in MGE-derived cells, and is associated with the differentiation of MGE-derived precursors in mice (Cobos et al., 2007). Here we found that the expression of different isoforms of PAK3 increased during the maturation of MGE-derived interneurons (Figures 3B–D). The expression of PAK3 was efficiently silenced with by the specific shRNA transfected in non-neuronal cells (Figure S2). Sholl analysis on MGE-derived cells transfected at DIV1 and cultured with BDNF up to DIV6 revealed that PAK3 silencing had only weak effects on neurite branching (Figures 4H,I), suggesting that PAK3 has no major impact in this phase of neuronal maturation.

We extended the analysis to the scaffold proteins ERC1/ELKS and liprin-α1 that are coexpressed in MGE-derived GABAergic interneurons (Figure 3B), and may interact with the PIX/GIT complexes (Figure 3A) (Astro and de Curtis, 2015). Test with respective shRNAs showed close to complete silencing of liprin-α1 and ERC1 in non-neuronal cells (Figure S2). Interestingly, silencing of either protein in MGE-derived cells showed strong impairment of neurite branching in DIV6 interneurons (Figures 4H,J). Overall the results highlight the importance of the network including the PIX, GIT, liprin-α1 and ERC1 proteins in the development of neurites during the development of MGE-derived GABAergic interneurons.

### GIT1 promotes neurite development by interacting with PIX and C-terminal binding partners

**Figure 5:**
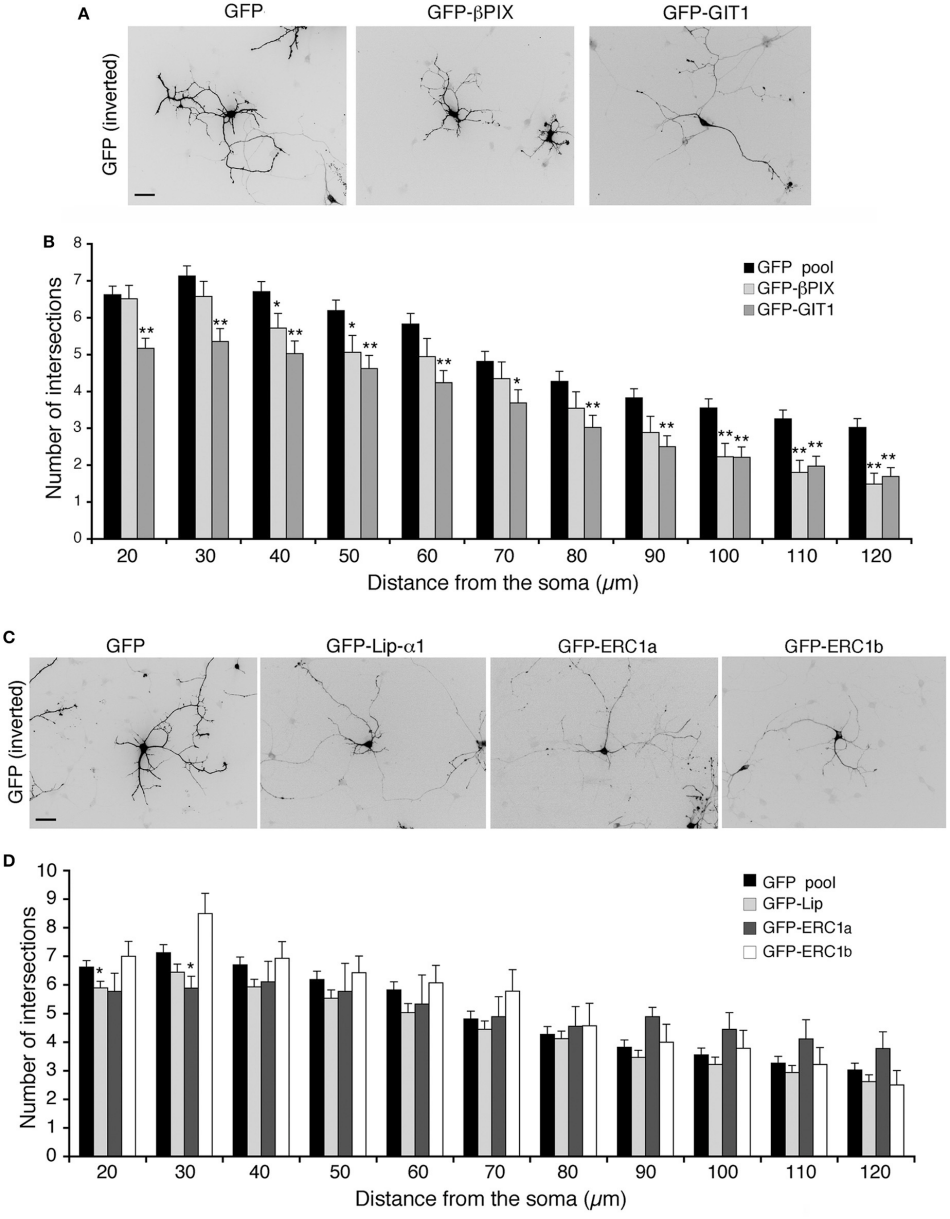
**Overexpression of full-length GIT1 inhibits neurite branching**. **(A,C)** MGE-derived interneurons expressing GFP, GFP-βPIX or GFP-GIT1 **(A)**, GFP, GFP-Liprin-α1, GFP-ERC1a (ubiquitous isoform), GFP-ERC1b (neuron-specific isoform) **(C)**, fixed at DIV6, immunostained for GFP. Scale bar, 40 μm. **(B,D)** Quantification of neurite branching by Sholl analysis. Bars are mean number of intersections ± SEM; *n* = 85 (GFP), 35 (βPIX 3), 42 (GIT1), 60 for GFP-Liprin-α1, 9 for ERC1a, 14 for ERC1b. ^*^p < 0.05; ^**^p < 0.01.

We have further investigated the role of the Rac–linked network by testing the effects of the overexpression of its components on the maturation of the neuritic tree. Sholl analysis revealed that βPIX overexpression affected the branching of neurites at longer distances from the soma (100–120 μm), while overexpression of GIT1 induced a more pronounced inhibitory effect on branching (Figures 5A,B). No consequences were observed after overexpression of either liprin-α1, or either one of two distinct isoforms of ERC1: the widely expressed ERC1a/ELKSε and the brain-specific ERC1b isoform (Wang et al., 2002; Astro et al., 2014) (Figures 5C,D). Therefore, only GIT1 exerts strong negative effects when overexpressed in the developing interneurons.

**Figure 6:**
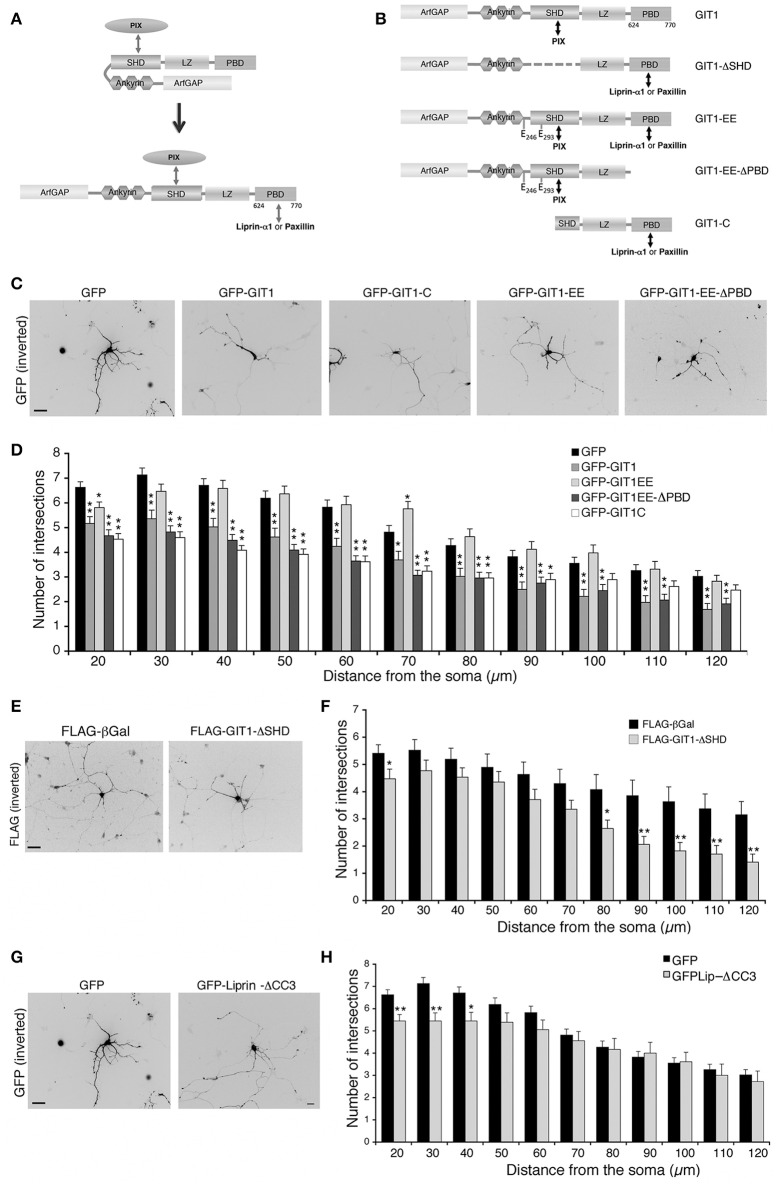
**Disrupting the interaction of GIT1 with either PIX or paxillin and liprin-α1 inhibits neuritic development. (A)** GIT1 autoinhibited (left) or active form (right) able to bind either paxillin or liprin-α1. **(B)** GIT1 mutants: double arrows indicate interaction with protein partners. **(C–H)** MGE-derived interneurons were transfected to express the indicated GFP- **(C,G)** or FLAG-tagged **(E)** proteins, fixed at DIV6 and immunostained for GFP or FLAG. Scale bars, 40 μm. **(D,F,H)** Quantification of neurite branching by Sholl analysis: bars are means ± SEM (*n* = 85 neurons for GFP, 42 for GIT1, 47 for GIT1-C, 41 for GIT1-EE, 45 for GIT1-EE-ΔPBD, 17 for GIT1-ΔSHD, 18 neurons for GFP-Liprin-ΔCC3). ^*^p < 0.05; ^**^p < 0.01.

In cells and brain lysates GIT1 is stably associated to βPIX (Totaro et al., 2007, 2012) that binds via the SHD (Spa2 homology domain) (Zhao et al., 2000). On the other hand binding of paxillin or liprin-α1 to the C-terminal region of GIT1 requires its activation by the release of an intramolecular interaction between the amino-terminal and C-terminal portions that keeps GIT1 in a binding-incompetent state (Totaro et al., 2007, 2012). Once the C-terminal binding region becomes available (Figure 6A), either paxillin or liprin-α1 can bind in a competitive manner to the C-terminal portion of the GIT1 polypeptide (Asperti et al., 2011). GIT1 is mainly found in the inactive form within cells (Totaro et al., 2007, 2012); the finding that not only GIT1 depletion, but also GIT1 overexpression inhibited neurite branching led us to speculate that the excess of the inactive full length GIT1 may interfere with the function of the endogenous PIX/GIT1 complexes required for normal neurite development (Figure 4). To test this hypothesis we made use of a number of GIT1 point and deletion mutants, in which the interactions with different partners and/or the activation state of the GIT1 protein were altered (Figures 6A,B). The autoinhibitory intramolecular binding between the N-terminal and C-terminal regions of GIT1 preventing binding to paxillin or liprin-α1 is abolished in the GIT1-EE mutant (Figure 6B). The substitution of two aromatic residues (tyrosines 246 and 293) with two glutamic acids near/at the SHD domain respectively makes the GIT1-EE mutant protein constitutively able to interact with paxillin or liprin-α1 (Totaro et al., 2014) (Figure 6A). On the other hand the mutant GIT1-C missing the N-terminal region can interact with paxillin or liprin-α1, but not with βPIX (Di Cesare et al., 2000; Ko et al., 2003). Sholl analysis showed that GIT1-C transfected interneurons had reduced neurite branching, while the expression of the constitutively active GIT1-EE mutant had normal neurite branching (Figures 6C,D). Expression of the GIT1-EE-ΔPBD deletion mutant that binds PIX, but lacks the C-terminal paxillin binding domain (PBD) resulted in the inhibition of neurite branching (Figures 6C,D). Therefore, the ability of the active mutant GIT1-EE to support neurite branching is dependent on binding to paxillin. The importance of GIT1–PIX complex was supported by the finding that GIT1-ΔSHD unable to bind PIX but able to bind paxillin and liprin-α1 (Totaro et al., 2007), had also an inhibitory effect on neurite branching (Figures 6E,F). The lack of effects observed in GIT1-EE transfected neurons may be explained by the fact the active mutant can interact with all partners required for neurite maturation: PIX, paxillin, and liprin-α1.

We next asked if the interaction between GIT1 and liprin-α1 is important for neurite extension. A central fragment of liprin-α1 (residues 513–673) interacts with an extended C-terminal region of GIT1 including the PBD (residues 523–770) (Ko et al., 2003; Asperti et al., 2011). Expression in GABAergic interneurons of GFP-Liprin-ΔCC3, a deletion mutant interacting poorly with GIT1 (Asperti et al., 2011), induced only a very mild reduction of neurite branching (Figures 6G,H). This mutant is able to interact efficiently with other liprin-α1 partners; thus the lack of a negative effect on branching suggests that the interaction of liprin-α1 with GIT1 is not essential for the proper development of neurites by MGE-derived interneurons.

Altogether these findings suggest that the interactions of GIT1 with PIX proteins on one side, and with the C-terminal partner paxillin on the other side are necessary for normal neurite development in MGE-derived interneurons.

### GIT1 and βPIX influence the morphology of growth cones

**Figure 7:**
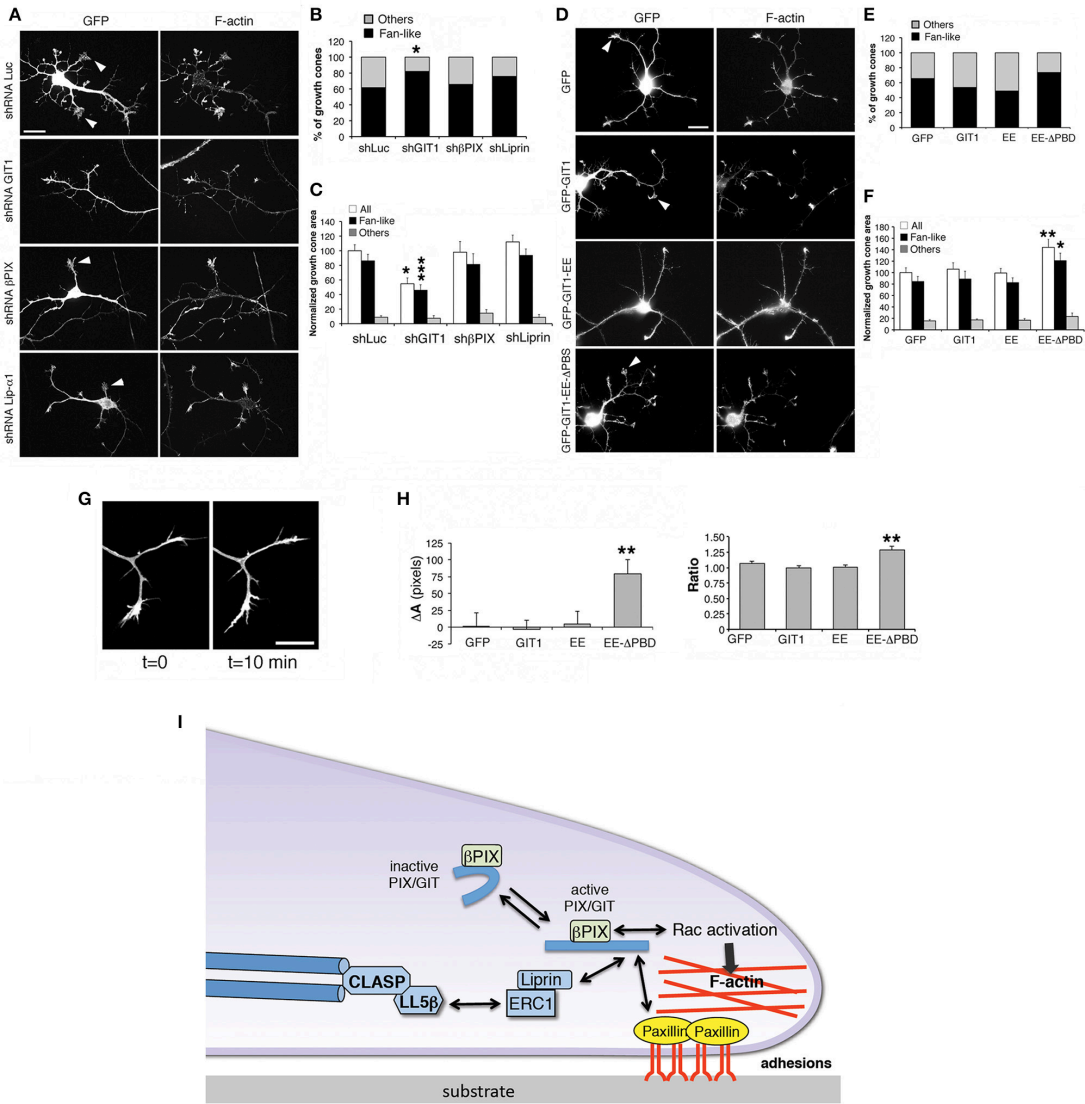
**GIT1 influences the shape of growth cones in developing interneurons**. **(A,D)** Representative images of DIV6 interneurons transfected either with shRNAs **(A)**, or with GFP-tagged GIT1 constructs **(D)** were fixed at DIV6 and immunostained for GFP and actin filaments. Arrowheads point to examples of fan-shaped growth cones. Scale bars, 20 μm. **(B,E)** Quantification of the percentage of fan-like vs. other types of growth cone morphologies in neurons transfected with shRNAs **(B)** or GFP-tagged GIT1 constructs **(E)**; *n* = 26–110 growth cones per experimental condition. ^*^p < 0.05; two-tailed χ^2^ Fisher-Yates test. **(C,F)** Effects of silencing **(C)** or protein expression **(F)** on the area of growth cones. Bars are means ± SEM. ^*^p < 0.05; ^**^p < 0.01; ^***^p < 0.001; two-tailed Student's *t*-test (*n* = 26–110 growth cones per experimental condition). **(G,H)** Dynamic analysis of growth cones. **(G)** Two frames from a time-lapse of growth cones from a DIV6 interneuron. Scale bar, 20 μm. **(H)** Quantification of the dynamic behavior of growth cones from interneurons transfected with indicated GFP-tagged constructs. Left graph: difference in area between two frames (at *t* = 10 min and *t* = 0 min). Right graph: ratio between the two areas at *t* = 10 min and *t* = 0 min (*n* = 19–35 growth cones from 10 to 11 neurons per experimental condition). ^**^p < 0.01. **(I)** Model for the function of the GIT1/βPIX complex at growth cones. Once activated, the GIT1/βPIX complex can bind to either paxillin or liprin-α1, two regulators of integrin-mediated adhesions to the extracellular matrix. The localization of the GIT1/βPIX complex near the cell membrane may also activate Rac GTPases, promoters of actin dynamics. This protein network may also be linked to microtubules via the LL5β/CLASP proteins (Lansbergen et al., 2006).

Neurite extension is driven by growth cones whose morphology and motility are based on filamentous actin dynamics (Lowery and Van Vactor, 2009). Analysis of growth cones in living cells revealed that their behavior matched with different growth cone morphologies, with the fan-like morphology typical of dynamic, searching behavior (Mason and Wang, 1997). We evaluated the effects of βPIX, GIT1 and liprin-α1 on the growth cones of interneurons during neurite development (Figure 7A). We found that only silencing of GIT1 increased significantly the proportion of fan-shaped growth cones compared to control neurons (Figure 7B), and decreased the average growth cone area (Figure 7C). We extended the analysis to full length and activated GIT1 mutants (Figure 7D). While expression of GIT1 or GIT1-EE did not produce evident effects on fan-shaped growth cones, the GIT1-EE-ΔPBD mutant unable to bind liprin-α1 or paxillin increased the size of growth cones (Figures 7E,F). We therefore tested the effects of GIT1-EE-ΔPBD on growth cones dynamics in living interneurons. We observed an increased variability in the morphology of growth cones from cells transfected with GFP-GIT1-EE-ΔPBD when compared to the growth cones from interneurons expressing GFP, GFP-GIT1, or GFP-GIT1-EE (Figures 7G–I). The effects on growth cones by the GIT1-EE-ΔPBD mutant may be due to the disruption of the interaction with the GIT1 C-terminal-binding partners paxillin and liprin-α1, which are involved in the regulation of cell adhesion and migration (Deakin and Turner, 2008; de Curtis, 2011).

To test if either GIT1 depletion or the expression of the GIT1-EE-ΔPBD mutant affected the distribution of proteins regulating the actin cytoskeleton, several actin regulatory proteins were examined in interneurons transfected with shGIT1 (shLuc as control) or GIT1-EE-ΔPBD (GFP as control). Among the proteins tested (including Src, active phospho-Src, cofilin, inactive phospho-cofilin, cortactin, N-WASP and VASP), only phospho-Src (active Src phosphorylated in tyrosine 416) showed a clear increase of the fluorescent signal in GIT1–depleted cells (Figure S4). No effects were observed by this analysis for any of the tested molecules in cells transfected with GIT1-EE-ΔPBD (data not shown).

## Discussion

In this study we have described the characterization of primary cultures derived from dissociated mouse embryonic MGE. Optimization of the culture conditions results in virtually pure GABAergic interneurons that have well developed dendrites and axons, and can form morphologically distinguishable inhibitory GABAergic synapses. To test the value of these cultures for the analysis of the intracellular mechanisms that drive interneuron differentiation, we have used the MGE-derived cultures to analyze the function played by a protein network implicated in the regulation of cell adhesion and motility, and expressed in GABAergic cells during neuritogenesis *in vitro*.

Embryonic MGEs contain precursors for a large fraction of the cortical and hippocampal GABAergic cells (Morozov et al., 2006; Wonders and Anderson, 2006; Tricoire et al., 2011). Substrates coated with PLL and laminin-1 were optimal for culturing the dissociated cells, and resulted in a dense network of neurons that express GABAergic markers already at DIV6. We found that MGE-derived cells mature into GABAergic interneurons with polarized morphology characterized by dendrites and one axon. Moreover, MGE-derived cells cultured for longer times either on PLL and laminin or on Matrigel formed morphologically-identifiable gephyrin- and VGAT-positive inhibitory synapses (Figure 1E). Although short-term MGE-derived cultures have been used (Cobos et al., 2007), to our knowledge this is the first study that has characterized in details this type of cultures, describing conditions that allow weeks-long cultures of primary murine cortical/hippocampal interneurons.

One advantage of using these virtually pure interneurons compared to mixed cortical or hippocampal cultures, is the possibility to perform biochemical analysis to determine types and levels of proteins expressed by GABAergic cells at different stages of differentiation *in vitro*. The immunochemical analysis of these cultures has allowed the identification of components of a protein network linked to Rac GTPases, which includes the scaffold proteins GIT, their interacting partners liprin-α1 and PIX, and the liprin-α1 partner ERC1. Interestingly, most proteins were upregulated with time in culture, suggesting their implication in the development of these cells.

The MGE-derived cultures described here will allow to comparatively analyze interneurons from many available transgenic/KO mice, which are relevant to the understanding of the function of cortical/hippocampal GABAergic cells. An additional advantage is the possibility to exploit the simplified *in vitro* system to look at processes independent from other types of neurons. In this direction, we have addressed the role of Rac GTPases in the development of these cells. Based on recent findings that Rac's are essential players for the correct development of cortical and hippocampal GABAergic cells *in vivo* (Vaghi et al., 2014; Tivodar et al., 2015; Pennucci et al., 2016), we have confirmed here that depletion of the endogenous Rac‘s impairs the neuritogenesis of interneurons *in vitro*.

To expand the knowledge of the intrinsic mechanisms linked to Rac GTPases during GABAergic development, we have shown here that proteins of the PIX/GIT network expressed in MGE-derived cells are required for proper neuritic development. The implication of GIT, PIX, liprin-α1 and ERC1 in interneuron differentiation is novel, while previous studies have shown that PAK3 is upregulated in post-migratory MGE cortical interneurons, indicating a role of PAK3 in post-mitotic GABAergic cell differentiation (Cobos et al., 2007; Dai et al., 2014). We detected only a minor effect of PAK3 depletion on the development of neurites *in vitro*. It is interesting that the expression of different alternatively spliced isoforms of PAK3 (Kreis et al., 2008) is strongly increased with development *in vitro*. This suggests that PAK3 may have a more prominent effect at later stages of interneuron differentiation, including later neuritogenesis and/or synapse formation, as suggested by the established role of PAK3 in the formation of excitatory synapses (Boda et al., 2004; Kreis et al., 2007).

We have extended the analysis of the GIT/PIX complexes by looking at the effects of their overexpression. Interestingly, overexpression of GIT1 and βPIX had an inhibitory effect on the development of neurites, while no detectable effects were observed after liprin-α1 or ERC1 overexpression (Figure 5). Although it is not easy at this point to find an explanation for these differences, it has to be considered that these proteins have a complex structure and may interact with distinct sets of partners, resulting in functional complexity. We have started to address this issue by exploring the function of GIT1 with a number of mutants. For GIT1, one hypothesis is that the excess of overexpressed inactive GIT1 may affect the function of the complex by sequestering PIX into an inactive complex unable to bind other partners like paxillin or liprin-α1 (Figure 6A). This hypothesis is supported by the observation that overexpression of either the full length protein (expected to be largely inactive: Totaro et al., 2007) or truncated mutants with defects in binding to specific partners (such as GIT1-EE-ΔPBD or GIT1-ΔSHD) has negative effects on neuritogenesis; while the overexpression of active GIT1-EE that can bind both PIX and the C-terminal partners, does not inhibit neurite extension.

A growth cone, similar to the front of a migrating cell, requires the turnover of the adhesions to the substrate and the dynamics of the actin cytoskeleton to drive motility and the consequent formation of a neurite. Based on our results we propose a model where GIT1 plays an important role as a scaffold to coordinate the activation of GTPase activity (via PIX proteins) and the dynamics of adhesions to the extracellular matrix (Figure 7I). For this, active GIT1 can interact either with paxillin, an adaptor protein that is part of the integrin-mediated adhesions to the extracellular matrix (Iwamoto and Calderwood, 2015), or with liprin-α1 (Asperti et al., 2011), an important regulator together with ERC1 of the turnover of integrin-mediated adhesions (Astro et al., 2016). According to the proposed model the functional interactions among these players are important to regulate the dynamic behavior of the growth cones. It is therefore not surprising that altering the levels of expression of GIT1 and βPIX proteins (either by silencing or by overexpression) may negatively influence the behavior of this protein network, resulting in the inhibition of neurite extension.

Interestingly, we observed an increase in active phospho-Src in cells depleted for GIT1. Src has been implicated in the regulation of the cytoskeleton and adhesion of cortical neurons during development: the increase in phospho-Src induced by Reelin, a factor promoting cortical radial migration, was accompanied by enhanced inhibitory phosphorylation of cofilin on the serine 3 residue (Chai et al., 2009). On the other hand, the expression of constitutively active Src in cortical neurons induced a decrease of the inhibited phospho-cofilin, while a dominant negative form of Src led to abnormal elongation of the leading processes in migrating neurons (Wang et al., 2015). How GIT1 and Src modulate the dynamics of growth cones during interneuron differentiations remains to be addressed.

In conclusion, we have shown here that MGE-derived cells may develop into polarized differentiated GABAergic interneurons that form morphologically identifiable inhibitory synapses. This culture system consisting of virtually pure GABAergic cells represents a valuable *in vitro* experimental setting for the biochemical and functional analysis of the intrinsic molecular mechanisms driving the development of GABAergic interneurons; it may be useful for relatively fast screenings of molecules relevant to the development of cortical and hippocampal interneurons *in vivo*; may represent an *in vitro* system for the functional analysis of molecules associated to pathologies in which interneurons are implicated; may be used for reconstitution experiments *in vitro*, by adding excitatory neurons from mice with different genetic background to study the contribution of either cell type to the phenotype of interest. Finally, the molecular analysis performed with these cultures may identify proteins and pathways relevant to understand the degree of differentiation of GABAergic interneurons derived from IPSCs.

## Materials and methods

### Animals

MGE-derived cultures were obtained from C57BL/6J (WT), Rac1^F/F^ (Rac1^flox/flox^), or Rac1^F/F^//Rac3^KO^ mice, generated as described before (Corbetta et al., 2005, 2009). For double deletion of Rac1 and Rac3, primary cultures from Rac1^F/F^//Rac3^KO^ mice were transfected with a pEGFP-Cre plasmid to inactivate the Rac1 gene *in vitro* (Corbetta et al., 2009). Experiments were carried out in accordance to the guidelines of San Raffaele Scientific Institute, in full compliance with national (D.L. n 116, G.U. suppl. 40, 1992 February 18, circular Nr. 8, G.U. 1994 July 14) and international agreements (EEC Council directive 86/609, OJ L 358, 1 DEC 12, 1987; NIH Guide for the Care and use of Laboratory Animals, U.S. National Research Council, 1996).

### Antibodies

Primary antibodies (Abs) used for immunofluorescence included: rabbit polyclonal Abs raised against αPIX (ARHGEF6 from Sigma), βPIX (si255 described previously; (Paris et al., 2003; Za et al., 2006)), GABA (Sigma), GFP (Invitrogen), MAP2 (Santa Cruz), VGAT (Synaptic System), FLAG (Sigma), anti-PAK1 (crossreactive with PAK2 and PAK3, from Santa Cruz), liprin-α1 and anti-Rac3 (Proteintech), phospho-Src (Tyr416; Cell Signaling), phospho-Cofilin 1 (Serine 3; Santa Cruz), N-WASP (kindly provided by Rajat Rohatgi); goat pAb anti-βPAK/PAK3 (Santa Cruz); chicken pAb anti-GFP (Abcam); mAbs raised against Cre (mouse IgG_1_; Covance), GAD67 (IgG_2a_; Millipore), gephyrin (IgG_1_; Synaptic System), PKL/GIT (IgG_1_ recognizing both GIT1 and GIT2; BD Bioscience), MAP2 (IgG_1_; (Sigma), tau-1 (IgG_2a_; Millipore), TuJ1 (IgG_2a_; Covance), tubulin (Sigma), Rac1 (recognizing both Rac1 and Rac3, from BD Bioscience), HA (Primm Biotech), ERC1 (IgG_2a_ ELKS-30 from Abcam), FLAG (IgG_1_; Sigma), Calbindin (from Swant), somatostatin (rat IgG2b; Millipore), cortactin (mouse IgG1; Millipore), VASP (9A2 rabbit mAb; Cell Signaling), cofilin (mouse mAb22 kindly provided by Hiroshi Abe, Bamburg laboratory), Src (mouse mAb; kindly provided by Margaret Jones, EMBL). Alexa Fluor A488-, 568-, and 647-conjugated secondary Abs were from Thermo Scientific, TRITC-conjugated phalloidin from Sigma. HRP-conjugated secondary Abs were: donkey anti-rabbit IgG and sheep anti-mouse IgG (GE Healthcare); rabbit anti-goat IgG (Southern Biotech).

### Plasmids and shRNAs

The pEGFP-N1 (Clontech Laboratories) and pFlag-LacZ plasmids were used as controls. The RFP-LifeAct plasmid used for live imaging was commercially available (Clontech Laboratories). The pEGFP-Cre was obtained as described (Rico et al., 2004). Plasmids pEGFP-GIT1 (for full-length human GIT1) was kindly provided by dr. Alan F. Horwitz (Schmalzigaug et al., 2007). Plasmids coding for GFP-GIT1-C (carboxy-terminal 347–740 residues of chicken GIT1), FLAG-GIT1-ΔSHD lacking the PIX-interacting region (deletion of residues 258–346 of chicken GIT1), GFP-GIT1-EE (activated form of full length human GIT1 with two substitutions: Y246E/Y293E), GFP-GIT1-Y246E/Y293E-ΔPBD (activated form of human GIT1 lacking residues 624–770 required for the interaction with paxillin and liprin-α1) were obtained as described (Albertinazzi et al., 2003; Za et al., 2006; Totaro et al., 2014). Plasmids coding for GFP-Liprin-α1 and GFP-Liprin-α1-ΔCC3 with deletion of residues 615–673 required for efficient binding to GIT1 were produced as described (Asperti et al., 2009, 2011). The plasmid for mouse GFP-βPIX was provided by Eunjoon Kim (Park et al., 2003). The plasmids for mouse GFP-ERC1a (mELKSε) and GFP-ERC1b (mELKSα) were provided by Ohtsuka et al. (2002). The plasmid for GFP-PAK3a was as described (Rousseau et al., 2003; Kreis et al., 2008). The plasmid for mouse HA-αPIX (Arhgef6) was kindly provided by Kerstin Kutsche (Kutsche and Gal, 2001).

DNA sequences for shRNAs were inserted into pSUPER–GFP plasmid (VEC-PBS-0005 from Oligo Engine) after digestion with Bgl II and Hind III according to the manufacturer's instructions. Target sequences were: shLuc (luciferase), CATCACGTACGCGGAATAC; shScr (scrambled), GAAATACCCACGAACTTTA; shGIT1, GCACTCAGCAACCGGCTCT; shGIT2, GAGTACTCCTCGACACGAA; shβPIX.1 CAACAGGAATGACAATCAC; shαPIX, GTCACCAGTTGATAGTAAA; shPAK3, GCAGCACATCAGTCGAATA; shβPIX.2, GAGCTGGAGAGACACATGG (Invitrogen); shLiprin-α1, GCTGGATGCTATCAACAAA (Invitrogen); shERC1, GAAGGAAGTATTAAGAGAA (Invitrogen). The targeting sequences for GIT1, GIT2, βPIX and αPIX.1 and PAK3 were derived from previously described siRNAs or shRNAs (Boda et al., 2004; Zhang et al., 2005; Nodé-Langlois et al., 2006; Heckel et al., 2009; Gavina et al., 2010; Totaro et al., 2012). Silencing by the different shRNAs was validated in transfected COS7 or in NIH-3T3 cells.

### MGE-derived cultures and transfection

MGEs harvested from E14.5 mouse embryos were dissected in cold L15 and maintained in ice cold HBSS with HEPES (Life Technologies). Tissue was enzymatically dissociated for 15 min at 37°C in 3 ml of HBSS with HEPES containing 0.05% trypsin and 100 μg/ml DNAse. Enzymatic digestion was stopped by addition of 5 ml of Plating Medium (Neurobasal medium, 2% B27 supplement, 1% GlutaMAX, and 10% fetal bovine serum, Thermo Scientific). Dissociated cells were centrifuged (5 min at 90 *g*), and the pellet was resuspended in 2 ml (3–6 embryos) or 3 ml (7 or more embryos) of Maintenance Medium (Plating Medium without serum). Cells were subjected to gentle mechanical dissociation and plated on 13 mm glass coverslips coated with 200 μg/ml poly–L-lysine (Sigma) and 20 μg/ml laminin-1 (Thermo Scientific).

Dissociated cells were cultured on different substrates to look for optimal conditions for the maturation of interneurons. Coating of coverslips included fibronectin (2.5 μg/ml), collagen I (50 μg/ml), collagen IV (20 and 40 μg/ml), laminin (20 and 40 μg/ml), PLL, 200 μg/ml, PLL and laminin-1 (200 μg/ml PLL coated with 20 μg/ml laminin-1), and Matrigel^TM^ (1.25 mg/ml). For cultures up to DIV15-DIV20 (no BDNF) cells were plated on 2.5 mg/ml of Matrigel^TM^. Cells were fixed after different times in culture and analyzed as described in the Results.

For transfection, cells were plated at high density (120.000 cells/well) on coverslips coated with 200 μg/ml PLL and 20 μg/ml laminin-1, and maintained in culture at 37°C, 5% CO_2_. At DIV1 cells were transfected with 0.5 μl of Lipofectamine-2000 (Thermo Scientific) and 0.1 μg DNA. Transfected cells were kept in maintenance medium with 50 ng/ml of BDNF (PeproTech) at 37°C, 5% CO_2_, and used at the indicated times.

### Cell lines and transfection

COS7 cells were cultured in Dulbecco's Modified Eagle's Medium containing 4.5 g/l glucose, 10% FetalClone III (Euroclone). NIH-3T3 cells were cultured in DMEM containing 4.5 g/l glucose, 10% Fetal Bovine Serum (FBS). Both cell types were transfected with Lipofectamine-2000 (Thermo Scientific), and incubated with 0.5 mg/ml G418 antibiotic (Calbiochem) to remove non-transfected cells. COS7 and NIH-3T3 cells were lysed for immunoblotting 2–4 days or 10–12 days after transfection, respectively.

### Lysates and immunoblotting

Cells, MGE-derived interneurons, E14.5 MGEs and P13 brains were lysed with 0.5% Triton X-100, 150 mM NaCl, 2 mM MgCl_2_, 20 mM Tris-HCl pH 7.5, 1 mM sodium orthovanadate (NaV), 10 mM sodium fluoride (NaF) and anti-proteases (1X Complete, Roche). P13 mouse brains were lysed in the same lysis buffer containing 1% Triton X-100. Lysates were clarified by centrifugation, and protein concentration was determined by the Bradford protein assay (BIO-RAD Laboratories). Lysates were subjected to SDS-PAGE on acrylamide gels and immunoblotting. Primary antibodies were detected with horseradish peroxidase-conjugated secondary antibodies and revealed by ECL (Amersham Biosciences).

### Immunofluorescence and image analysis

MGE-derived interneurons were fixed with 4% paraformaldehyde and processed for immunofluorescence after different times in culture (DIV1-DIV20). To characterize the maturation of MGE-derived interneurons *in vitro*, WT and Rac3KO primary cultures were fixed and immunostained for several neuronal and GABAergic markers including neurotransmitter GABA, TuJ1, calbindin, GAD67, somatostatin, MAP2 and tau-1. Images were acquired with a Zeiss Axiophot Microscope with a 20x lens equipped with a Hamamatsu C4742-95 digital camera. Images were processed with the public domain NIH software ImageJ (NIH; RRID: nif-0000-30467). The background was subtracted from each image before quantification. We then calculated the percentage of cells positive for each marker with respect to the total number of cells, identified by nuclear staining with DAPI.

Sholl analysis was used to quantify neurite branching in transfected interneurons, identified by staining with anti-GFP Abs. We used a mask consisting of eleven concentric circles of gradually increasing radius (between 20 and 120 μm), starting from the centroid of the cell body, and drawn with a dedicated ImageJ plugin. The number of intersections between the rings and the neurites was scored manually. Only protrusion with a length of at least 10 μm were considered as neurites/branches. The data were analyzed by the Student's *t*-test and by the two-way repeated-measures analysis of variance (ANOVA; transfection group and distance from the soma as factors) followed by the Bonferroni *post hoc* test for significance. Differences were considered statistically significant for *P* < 0.05.

For the analysis of growth cone morphology, images were acquired on a Perkin Elmer UltraVIEW Spinning Disk Confocal Microscope with a 63x NA 1.4 oil-immersion objective, or an Axio Observer.Z1 inverted microscope with a 63x NA 1.4 oil-immersion objective, equipped with an AxioCam MRm camera (Zeiss), and processed with ImageJ. The analysis was performed into two different steps: growth cones were first classified according to their shape (fan type or non-fan type), and the percentage of each type was calculated. In the second step, the projected area of each growth cone was quantified by ImageJ.

For the analysis of growth cone dynamics, time-lapse experiments were performed on DIV5-DIV7 MGE-derived interneurons. The behavior of growth cones was evaluated in neurons cotransfected with RFP-LifeAct and either GFP, GFP-GIT1, GFP-GIT1-EE, or GFP-GIT1-EE-ΔPBD. During the experiment the cells were maintained in a temperature and CO_2_-controlled chamber at 37°C. Images were acquired at 30 s intervals with a scanning confocal microscope (TCS SP8 SMD-FLIM, Leica) using a 63X 1.4 NA oil immersion objective. The dynamic behavior of growth cones was evaluated by comparing different parameters confronting two time points, *t* = 0 min and *t* = 10 min. This analysis was performed with ImageJ.

The distribution of proteins expressed in growth cones of MGE-derived interneurons was evaluated by confocal microscopy in cells transfected with shLuc, shGIT1, GFP or GFP-GIT1-EE-ΔPBD and immunostained for GFP, F-actin, cortactin, Src, phospho-Src (Tyr 416), cofilin, phospho-cofilin, N-WASP and VASP. Transfected GFP-positive interneurons were analyzed with ImageJ. The background was subtracted from each image before quantification. We calculated the intensity of fluorescence signal in growth cones normalized on the growth cone area. The data were analyzed by the Student's *t*-test; differences were considered statistically significant for *P* < 0.05.

## Author contributions

SF, VA, JB, and Id contributed to the conception and design of the work; SF, VA, RM, DT, and MB developed the experimental design; SF, VA, and Id wrote the manuscript.

## Funding

This work was supported by the Telethon Foundation, Italy (grant GGP12126) and by AIRC (grant 15530).

### Conflict of interest statement

The authors declare that the research was conducted in the absence of any commercial or financial relationships that could be construed as a potential conflict of interest.
