# Supplementary figures and images for: Identification of a Protein Network Driving Neuritogenesis of MGE-Derived GABAergic Interneurons
Source: Front Cell Neurosci. 2016 Dec 21;10:289. doi: 10.3389/fncel.2016.00289 (PMC5174131; doi:10.3389/fncel.2016.00289)

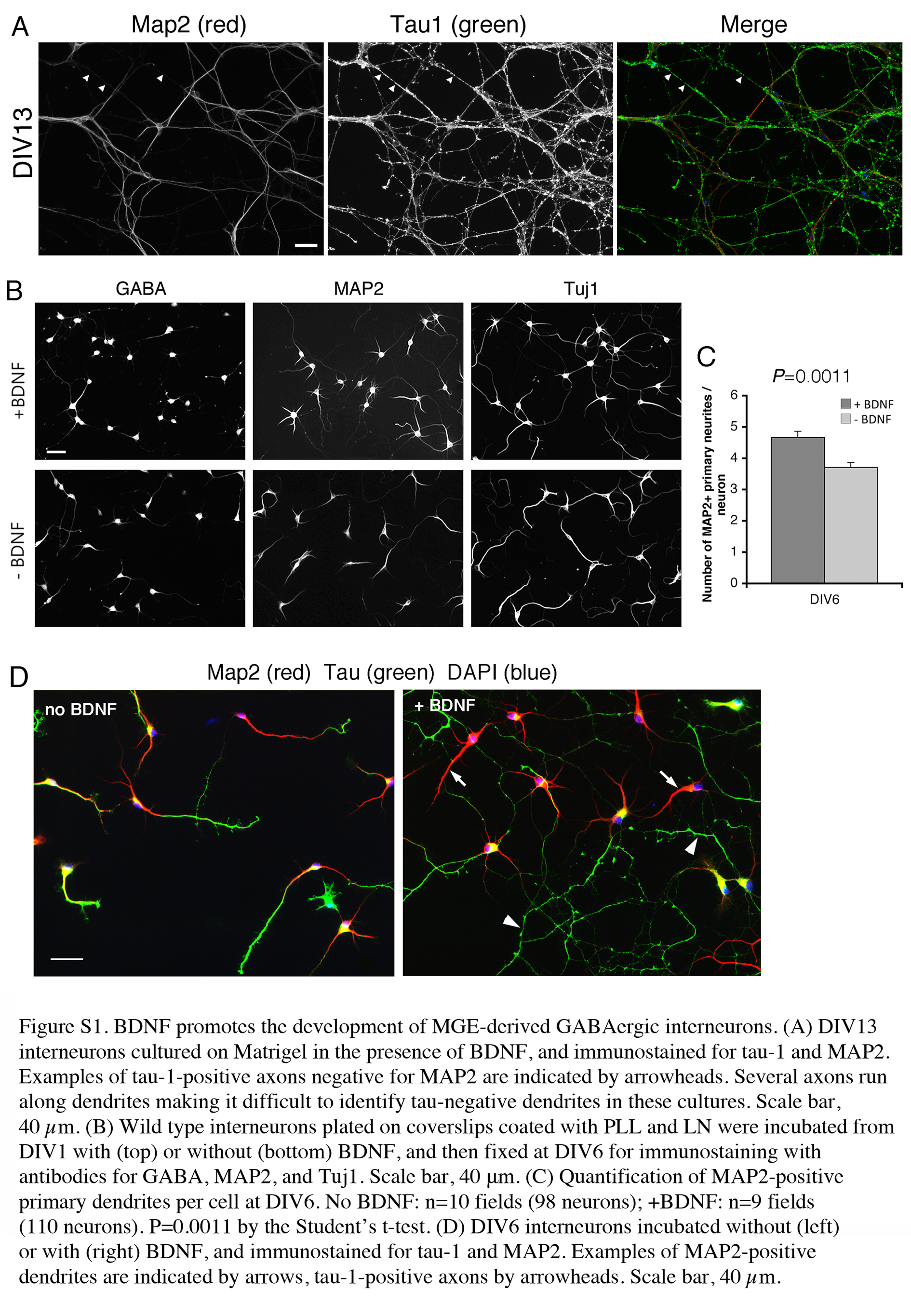

Supplement: Supplementary file 1 [file Image1.TIF]

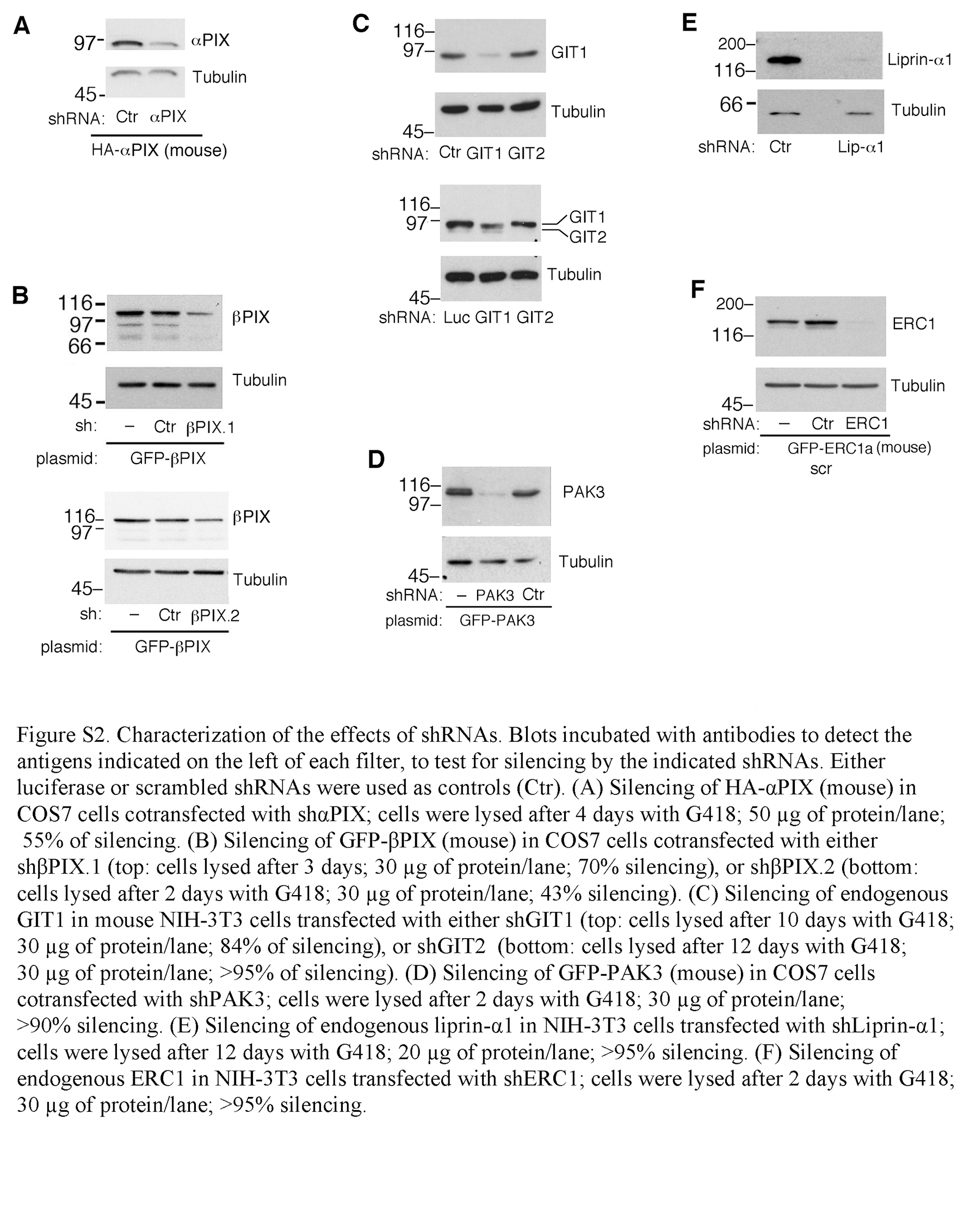

Supplement: Supplementary file 2 [file Image2.TIF]

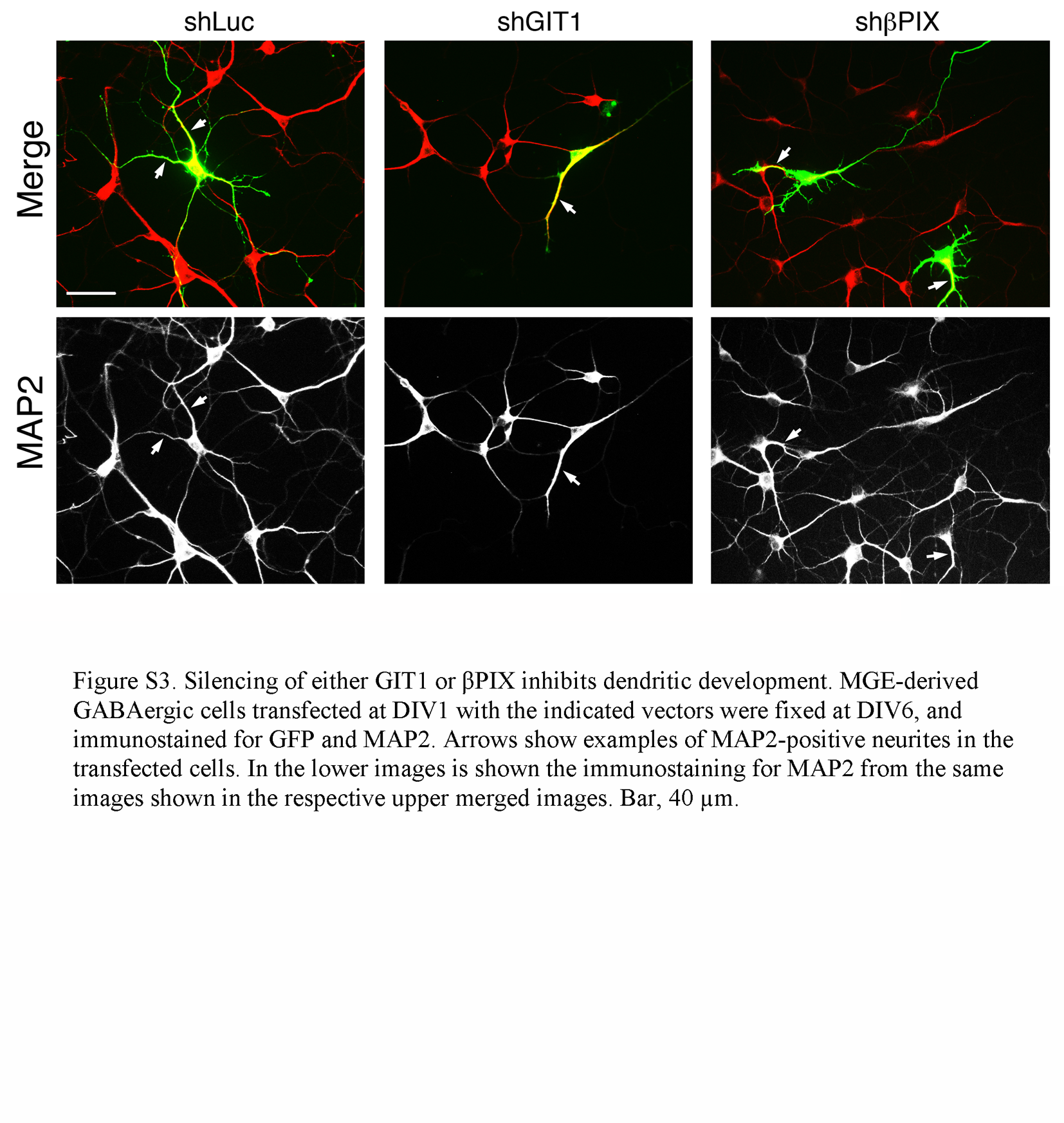

Supplement: Supplementary file 3 [file Image3.TIF]

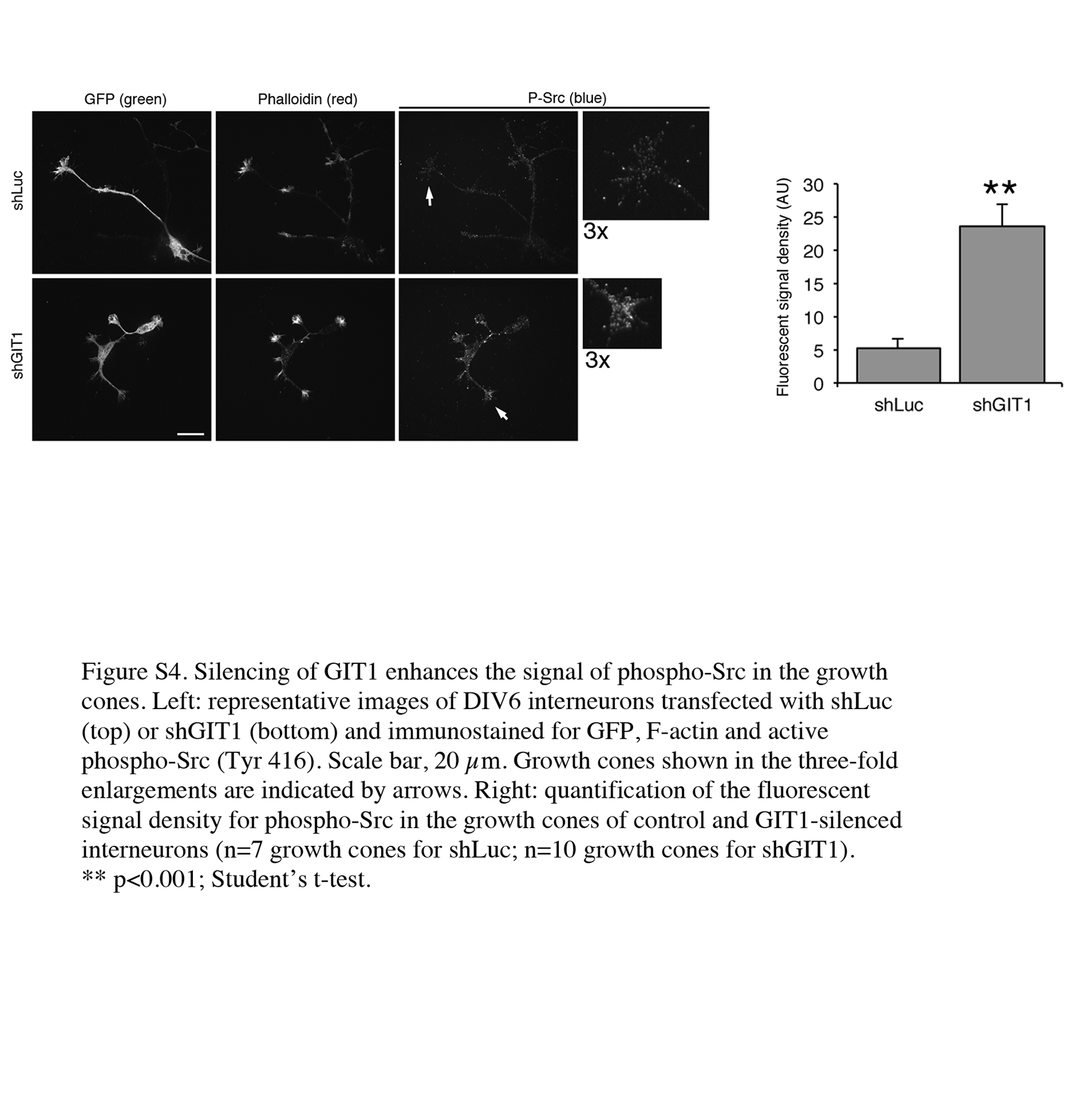

Supplement: Supplementary file 4 [file Image4.TIF]
